# First Design of a Contact Lens for Diagnosis of Dehydration

**DOI:** 10.3390/bios15080532

**Published:** 2025-08-14

**Authors:** Kundan Sivashanmugan, Reece E. Albert, Joseph R. Lakowicz

**Affiliations:** 1 Center for Fluorescence Spectroscopy, Department of Biochemistry and Molecular Biology, University of Maryland School of Medicine, 655 W. Baltimore St, Baltimore, MD 21201, USA; skundan@som.umaryland.edu; 2 Department of Obstetrics, Gynecology and Reproductive Sciences, University of Maryland School of Medicine, 655 W. Baltimore St, Baltimore, MD 21201, USA; areece@som.umaryland.edu

**Keywords:** dehydration, sodium/potassium ions, silicone hydrogel contact lens, tear, fluorescence, point-of-care diagnosis

## Abstract

Dehydration is a serious medical problem for elderly patients and young children. The most widely used diagnostics are measurements of sodium ion (Na^+^) and potassium ion (K^+^) in blood serum. Dehydration is difficult to diagnose even by trained health care professionals because the body compensates to maintain the appearance of skin. These measurements required a blood draw because specific tests are generally not available for only Na^+^ and K^+^. The blood samples are analyzed by an electrolyte panel (EP) or a basic metabolic panel (BMP). Most hospitals limit EP and BMP to one per day to control costs. More frequent measurements of Na^+^ and K^+^ are needed, especially during rehydration. We designed a dehydration contact lens that can provide the Na^+^ and K^+^ concentrations as needed or for continuous monitoring. The measurements are obtained from the fluorescent lifetime or wavelength-ratiometric intensities of the Na^+^- and K^+^-sensitive fluorophores. The dehydration contact lens does not contain electronic components and are inexpensive to prepare.

## 1. Introduction

Dehydration is a world-wide health problem, resulting in over 1.35 million deaths per year [1]. Approximately half of these deaths are due to dehydration with diarrhea, and many of these are due to poor water quality. Younger children <5 years of age and adults >65 years of age are the most affected populations. In the senior population, 5–10% of deaths are due to dehydration [2,3]. The percentage is possibly higher because deaths of the elderly are often attributed to broken bones, which can be the result of falling due to dehydration. In the general population, one out of five illnesses can be prevented by adequate hydration [4,5,6,7]. In developed countries dehydration occurs among the elderly, often those with other medical conditions. Elderly patients admitted to the hospital due to dehydration, without other medical conditions, have a 50% chance of dying [3], and those who recover can have cognitive deficiencies [8]. There are multiple reasons for dehydration in elderly individuals. With increasing age there is a decrease in the total body water for both men and women. Because of the smaller water volume, sodium concentration increases more rapidly with loss of water. The elderly have a decreased sense of thirst [9,10] and often do not drink enough fluids to replace a water deficiency. Additionally, the concentrating ability of the kidneys is decreased with aging. Elderly patients in nursing care homes are frequently dehydrated [7], and those admitted to hospitals have a significantly greater risk of in-hospital mortalities [10].

Dehydration is often not reported as a primary diagnosis because it occurs concurrently with many other medical conditions. Numerous reports have demonstrated that a physical examination of the patient is not a reliable way to identify dehydration [11,12,13,14]. Diagnosis of dehydration requires measurements of sodium ion (Na^+^) and potassium ion (K^+^) concentrations in blood, serum, or plasma [15]. These concentrations are typically determined from a standardized electrolyte panel (EP) or a basic metabolic profile (BMP) panel. A blood draw is necessary to conduct the EPs and BMPs, which are used to measure the concentrations of Na^+^, K^+^, and other analytes [15]. However, most hospitals only allow one blood draw per day, which may not be adequate for many patients with dehydration. The time delay to receive EPs or BMPs does not allow Na^+^ and K^+^ measurements at the bedside or during intravenous (IV) rehydration treatment. If too rapid IV rehydration can result in neurological damage [16,17,18]. Individual Na^+^ and K^+^ measurements are rarely requested or performed because the EP and BMP are fully automated. The limited number of panels creates a danger for patients with dysnatremia, or rapid changes in sodium concentrations, which can result in death or neurological problems [16,17,18]. Additionally, repeated venipunctures may lead to hospital-acquired anemia (HAA), particularly in frail or post-operative individuals, and result in higher hospitalization expenses.

Unfortunately, other methods to diagnose dehydration have not been effective. For children, diarrhea, vomiting, and weight loss have only modest value for assessing dehydration but are not useful for older individuals [19,20]. Using specific gravity or plasma osmolarity has almost no diagnostic value for older individuals [13]. As a result, other technologies have been tested to diagnose dehydration. The more recent literature describes new technologies, which include testing of sweat, saliva, bioimpedance over a wide range of frequencies, infrared absorption, and Raman spectroscopy [21,22,23]. None of these methods is able to diagnose dehydration and does not provide useful clinical information. There is no gold standard method to diagnose dehydration [16]. Therefore, a straightforward method for diagnosing dehydration is necessary for individuals of all age groups. Tear samples offer a non-invasive method, but their use is restricted by the total volume (7–10 µL) of tears available [24,25]. Larger tear volumes are not available because physical contact with the eye results in a rapid change in tear composition [24,25].

The first contact lenses were made of glass or a hard plastic methacrylate (PMMA) [26]. These hard lenses did not permit oxygen transport to the cornea, which limited the wear time. Contact lens technology was improved by the introduction of soft contact lenses made from hydrogels (HG), which improved oxygen permeability [26]. However, for adequate permeability, the HG polymer content had to be very low, and the HG lenses were too fragile for handling [26,27,28]. Silicone (not oxidized, SiO_2_) is known to be highly permeable to oxygen due to high oxygen diffusion and solubility [29,30,31,32]. Silicone itself is too hydrophobic to be used on the sensitive corneal cells and does not allow transport of tear electrolytes [26,27,28,29,30,31,32]. This resulted in extensive commercial research to develop silicone-containing precursors to create silicone hydrogel (SiHG) lenses with unique morphology (Figure S1). There are numerous reports describing smart contact lenses (SCLs) that contain electronic components for receiving and sending signals, sensing of the analyte, and an antenna for charging by electrical induction [22,33]. Even if some SCLs are successful, they are likely to be too expensive for medical applications, such as discarding after one test or one week. The SiHG lenses are distinct from HG lenses. SiHG lenses have continuous silicone channels for oxygen transport and open aqueous channels for transport of water and ions. This morphology contains both hydrophobic and hydrophilic regions and interface regions, which can be used to bind sensing fluorophores. Over the past several years we have developed methods to bind fluorophores to SiHG lenses [34,35,36,37,38]. One of the methods takes advantage of the hydrophobic region of SiHG lenses. Long-chain hydrocarbons are attached to ion-sensitive fluorophores, which bind by hydrophobic effects [34]. The second method, used in this report, takes advantage of the high affinity of poly-L-lysine (PL) for SiHG lenses. PL was selected because of the well-known binding of lysozyme to SiHG lenses, and lysozyme is probably the only positively charged protein in tears. Fluorophores linked to PL were found to bind quickly and strongly to several different SiHG lenses [34,35,36,37,38].

**Figure 1:**
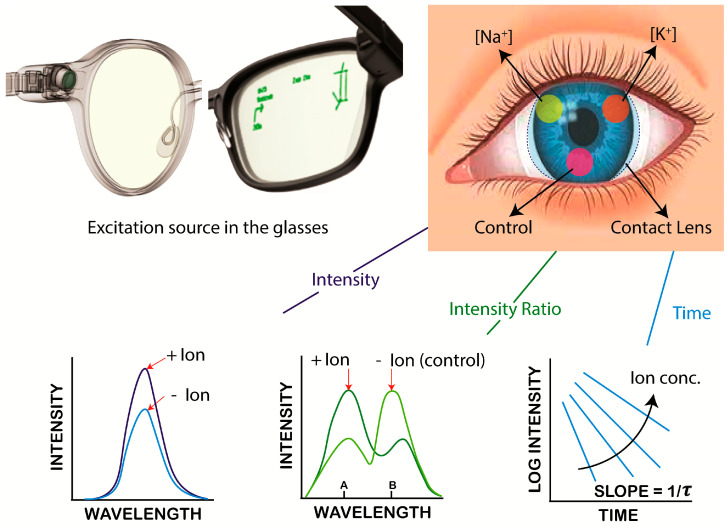
Schematic illustrating commercially available glasses with built with electrical and optical components and an image of the proposed dehydration contact lens. The bottom row illustrates the fluorescence-based methods employed to measure emission from labeled contact lenses.

In the present report we describe the synthesis of different Na^+^- or K^+^-sensitive fluorophores bound to PL. The lenses also contained rhodamine B (RhB), which was not sensitive to either ion, which was used for reference intensity. The ion concentrations were determined using fluorescence intensities, fluorescence intensity ratios, or lifetimes of the PL-linked fluorescent probes. Both Na^+^ and K^+^ measurements could be made in the physiological range of these ions (Table S1). The fluorescence intensity ratio at different emission peak wavelengths and lifetime measurements can be determined even if the fluorescence signals are unstable, as illustrated in Figure 1. These lenses can provide on-demand measurements of Na^+^ and K^+^ without the need for additional blood draws.

## 2. Materials and Methods

### 2.1. Selection of SiHG Contact Lens

In this work, for the purpose of developing sodium and potassium contact lenses (NaK-CLs), we selected two SiHG contact lenses that are easily obtainable in the market. The Comfilcon A (Biofinity, CooperVision, New York, NY, USA) and Lotrafilcon A (Air Optix, Alcon, TX, USA) contact lenses were chosen for testing because roughly 65% of new prescriptions advised by ophthalmologists contain these lens types, indicating their extensive use among the general public [39,40]. The second-generation SiHG lenses have high oxygen permeability, allowing them to be worn continuously for up to 30 days without causing pain. The Air Optix lenses probably contain more silicone than the Biofinity lenses (Figure S1), so the aqueous regions are shown to be narrower than for the Biofinity lenses, but precise 3D structures are not known. From the structures of starting monomers, the SiHG lenses appear to have a net negative charge and are known to become coated by the positively charged lysozyme in tears [34,35,36,37,38].

### 2.2. Synthesis of the Sodium-Sensitive and Potassium-Sensitive Fluorophores

**Figure 2:**
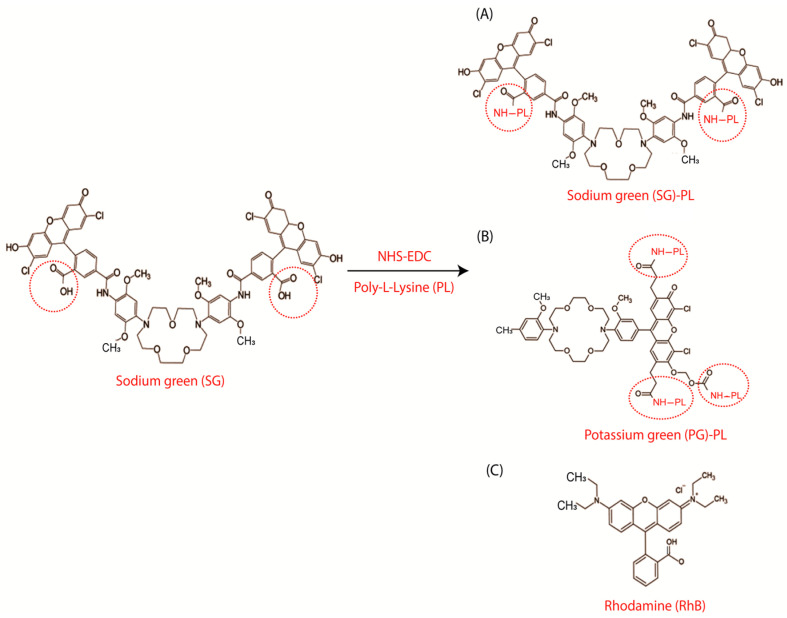
(**A**) Chemical synthesis and structure of sodium green poly-lysine (SG-PL), (**B**) potassium green poly-lysine (PG-PL), and (**C**) structure of the reference probe rhodamine B (RhB).

We synthesized sodium- and potassium-sensitive fluorophores by using Sodium Green (SG, tetramethylamonium salt, cell impermeant) and Potassium Green (PG, Potassium Green-2 Salt, cell impairment), as described in our initial materials in our previous work [36,37]. Both sodium- and potassium-sensitive probes were conjugated to the PL (Advanced Biomatrix, CA, USA) to allow the probes (SG or PG) to bind to contact lenses. The free carboxyl groups on the sodium and potassium probes were initially activated using the NHS/EDC method, and amide bonds were then formed using PL. Figure 2 shows the chemical structures of the probes and the synthesis procedures. More specifically, 1 mg of SG and PG were added to 2 mL of dimethylformamide (DMF, Sigma-Aldrich, Missouri, USA) in a solution that included 0.20 mL of 0.01% PL (MW 70–150 kDa). The SG and PG probe solutions were activated using N-hydroxysuccinimide (NHS, 0.27 mg in 2 mL (Sigma-Aldrich) and N-(3-dimethylaminopropyl)-N carbodiimide hydrochloride (EDC, 0.33 mg in 2 mL) for 1 h at room temperature and an inert atmosphere. After the addition of PL into the SG or PG probe, the solution was stirred continuously for an additional hour. Finally, the final SG-PL or PG-PL probe solutions were bound to the contact lens by diluting 0.020 mL aliquots of the prepared reaction mixture in 2 mL of water. In this study, the rhodamine B (RhB, Sigma-Aldrich) probe was used as a reference probe due to its ability to provide a signal that was independent of sodium and potassium concentrations. RhB was used without chemical modification because it spontaneously binds to the lenses. This result was expected because RhB was known to bind to cell membranes [41,42].

### 2.3. Probe Labeling of SiHG Lenses

**Figure 3:**
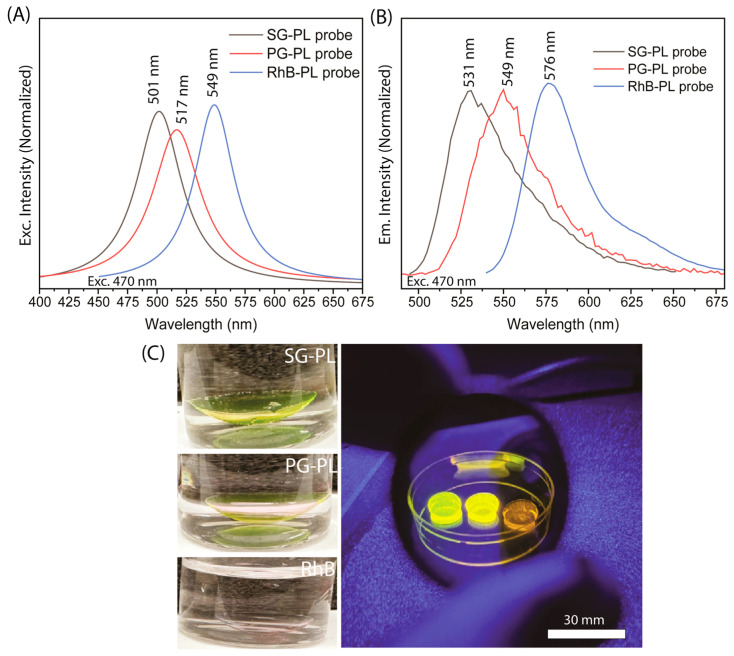
Excitation and emission spectra of SG-PL, PG-PL, and RhB probe solutions (**A**,**B**). The excitation and emission spectra were measured using 20 µL aliquots of the as-prepared reaction mixture diluted in 2 mL of water and measured at a wavelength of 470 nm. (**C**) Photographs of the lenses labeled with SG-PL, PG-PL, and RhB in Lot A lenses. Left, white images; and right, under a UV hand lamp.

A simple approach was used to attach an SG-PL or PG-PL probe onto Lot A or Con A lenses. A 20 µL solution of SG-PL probe was diluted with 2 mL of an aqueous methanol (50–50 v/v) solution in order to attain a probe concentration of 1 µM. Next, the mixture was thoroughly stirred and thereafter transferred to a cuvette size 1 cm × 1 cm, which was used to hold the lenses in a diagonal orientation for probe uptake. The Lot A or Con A lenses were incubated with a probe solution for about 90 min. Additionally, the lenses were subjected to a washing procedure with deionized water to eliminate any unattached probes. A similar method was used to analyze the uptake of PG-PL probes into lenses. In order to perform ion sensitivity measurements, lenses with SG-PL or PG-PL probes were used for fluorescence measurement (Figure 3A,B). The labeled lenses could be seen visually under a UV hand lamp (Figure 3C).

### 2.4. SiHG Lenses Fluorescence Measurements

Using fluorescence measurements, the SG-PL and PG-PL probes attached to contact lenses were tested for ion sensitivity. The lenses were positioned diagonally inside a 1 cm × 1 cm cuvette and covered with MOPS buffer at room temperature and pH 7.2. The SG-PL and PG-CL lenses were examined with varying concentrations of NaCl and KCl in 2 mL of buffer. The emission spectra and lifetime data were obtained for each concentration of sodium and potassium. The intensity and intensity decays of ion-sensitive SiHG lenses' fluorescence spectra were obtained using a FluoTime 300 instrument from PicoQuant, Berlin, Germany. The subsequent analysis was conducted using the EasyTau V2 software. A 480 nm laser diode was used as the excitation source, operating at a repetition rate of 40 MHz with a pulse width of less than 100 ps. The measurement of intensity decays was conducted at the emission maximum, and the analysis was conducted using the multi-exponential model and described previously [43,44]. The emission spectrum and lifetimes were measured at five different positions on each contact lens sample. The SG-PL or PG-PL were also examined using fluorescent lifetime imaging microscopy (FLIM) from ISS (Champaign, IL, USA). The FLIM technique [45,46] used a dual scanning capability, with a 473 nm laser diode as the excitation source. The excitation rate was set at 40 MHz, and the pulse width was around 250 ps. The contact lenses were imaged at a distance of 13 mm over the entire lens area, with a resolution of 256 × 256 pixels.

## 3. Results

Three fluorophores were selected for the dehydration contact lens. In each case the starting fluorophores were SG and PG contained free carboxyl groups that were linked to amino groups on PL. The reference probe rhodamine B (RhB) showed no observable interaction with PL-coated contact lenses and is known to be environmentally insensitive. Nonetheless, RhB was previously known to spontaneously bind to cell membranes [47]. The excitation and emission spectra of these probes showed considerable spectral overlap (Figure 3). We reasoned that selection of excitation and emission wavelengths and wavelength-dependent lifetime measurements would allow these three species to be independently quantified. SG-PL and PG-PL were rapidly bound to both the Con A and Lot A contact lenses, as could be observed visually (Figure 3C) and by fluorescence intensity measurements (Figure S2). Figure S3 shows that probe uptake occurs; however, it cannot be used to calculate accurate uptake rate constants because the incubation probe solution was depleted of free probes during measurement.

**Figure 4:**
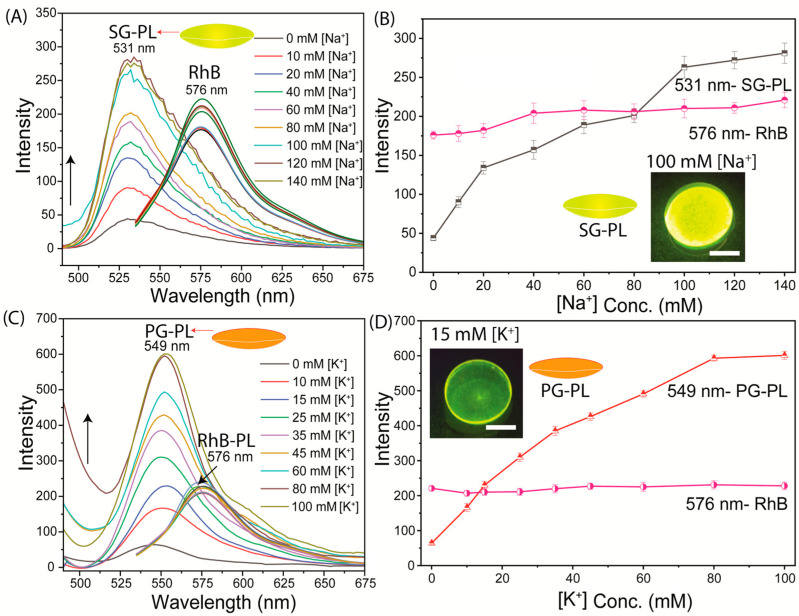
(**A**) Sodium response of SG-PL and RhB bound to two separate Lot A lenses. (**B**) Effects of sodium on the intensities of SG-PL at 531 nm with RhB in the Lot lens. (**C**) Potassium response of PG-PL and control RhB bound to two separate Lot A lenses. (**D**) Effects of potassium on PG-PL at 549 nm with RhB in the Lot lens. The insert emission photography shows a 100 mM Na^+^ lens (**C**,**D**) a 15 mM K^+^ lens, and the scale bar is 5 mm.

**Figure 5:**
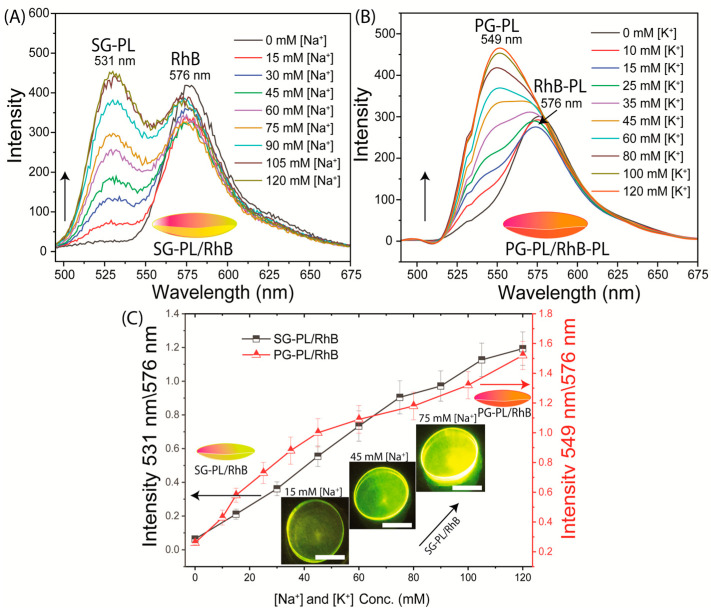
(**A**) SG-PL and RhB both bound to the same Lot A lenses. (**B**) PG-PL and RhB both bound to the same Lot A lenses. Wavelength-ratiometric measurements of SG-PL/RhB and PG-PL/RhB measurements with RhB as the reference fluorophore (**C**) (note, black line is associated with Na^+^ and red line is associated with K^+^). The insert emission photography shows a 15, 45, and 100 mM Na^+^ lens, and the scale bar is 5 mm.

The Na^+^ and K^+^ probes were tested for sensitivity to their target ions when bound to Lot A lenses (Figure 4 and Figure 5). The tests were performed in two ways. The first test was performed using SG-PL alone in the lens, with the RhB reference in a separate lens (Figure 4A). These tests showed that the SG-PL intensity increased by about 9-fold as the Na^+^ concentration increased, and there was no major impact on the RhB fluorescence. The second test was performed with both SG-PL and RhB bound to the same lens (Figure 5A). Essentially, the same results were found for the Lot A lens with both SG-PL and RhB bound to the same lens. This demonstrates that the RhB reference fluorophore had no effect on the SG-PL. A similar result was obtained for the K^+^ probe PG-PL when bound to the Lot A lenses (Figure 4C and Figure 5B). The results in Figure 4 and Figure 5 showed no effect of Na^+^ or K^+^ on the RhB emission.

To quantify the extent of ion binding, the ion-dependent intensities of SG-PL and PG-PL bound to lenses are shown in Figure 4B,D. These results show the probes are sensitive to their target ions in the physiological range (Table S1). Figure 5C shows the ion-dependent intensities relative to RhB. In the present case, with stable positioning of the lenses, the ratio measurements do not provide additional information. However, if the lens was being worn by a patient, there would be changes in the total intensities, and they could be accounted for by the wavelength-ratiometric measurements. NaK-CL lenses demonstrated sensitivity to Na^+^ and K^+^ within physiologically relevant ranges. Detailed linear fitting results are provided in Figure S4, which showed the fluorescence response to varying concentrations of Na^+^ (0–140 mM) and K^+^ (0–100 mM).

**Figure 6:**
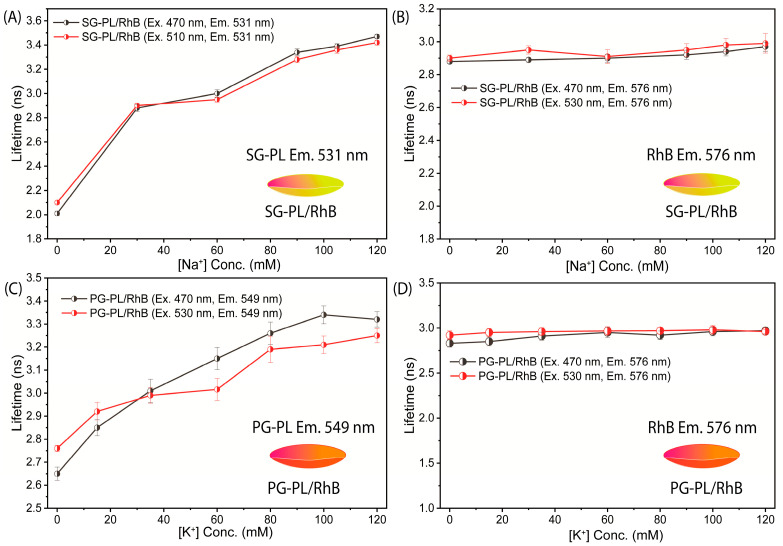
Fluorescence lifetime of an SG-PL/ RhB lens measured at the SG-PL emission maximum (**A**) and at the RhB emission maximum (**B**). Fluorescence lifetime of a PG-PL/RhB lens measured at the PG-PL emission maximum (**C**) and at RhB emission maximum (**D**).

**Figure 7:**
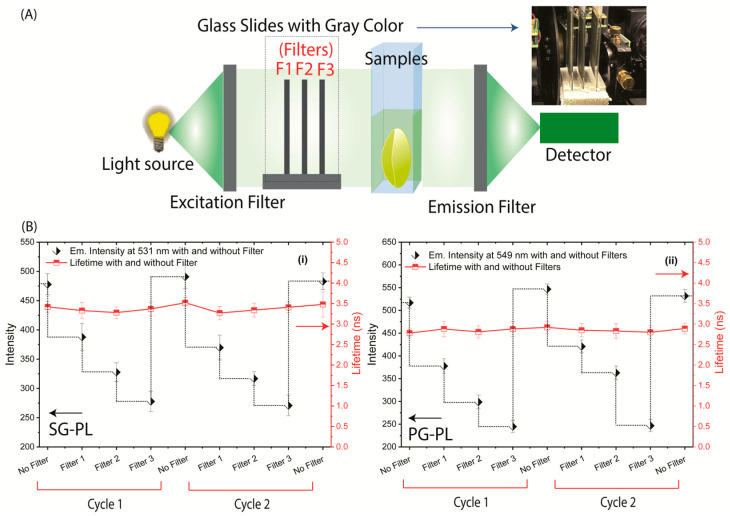
(**A**) Schematic diagram showing emission intensity and lifetime measurements using thin gray glass filters placed between the excitation source and the samples. Fluorescence intensities and lifetime SG-PL lenses with 0.0 to 130 mM Na^+^ (**B**(**i**,**ii**)) PG-PL lenses with 0.0 to 110 mM K^+^ for excitation intensity changed by thin gray glass filters between the source and samples. The emission intensities are changed by the filters, but the lifetimes remain constant.

The fluorescence lifetimes of SG-PL, PG-PL, and RhB bound to Lot A lenses over a range of ion concentrations are shown in Figure 6. Both SG-PL and PG-PL displayed a 9-fold increase in intensity with increased ion concentrations. However, SG-PL and PG-PL displayed only a 60% increase in lifetime from 0.0 to high concentrations of Na^+^ and K^+^. The lifetime of RhB was independent of the ion concentrations. The lifetime changes in SG-PL and PG-PL are smaller than expected when compared to the intensity changes (Figure 4 and Figure 5). At present we do not know the reason for this difference, but the probes demonstrate the possibility of lifetime-based sensing of Na^+^ and K^+^ in contact lenses. This fact is demonstrated in Figure 7, where the lifetimes of SG-PL and PG-PL were measured with different excitation intensities caused by placing neutral density filters into the excitation pathways. The lifetimes of these probes remained constant for 2-fold changes in excitation intensity.

**Figure 8:**
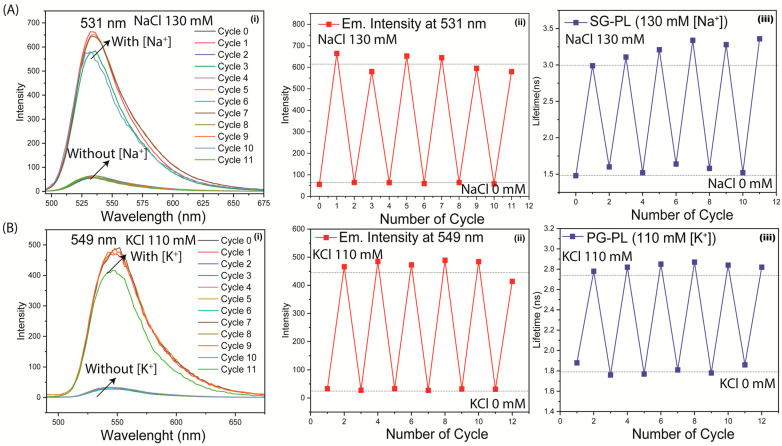
Reversibility of the response to different ion concentrations for SG-PL and Na^+^ (**A**(**i**–**iii**)), and PG-PL and K^+^ (**B**(**i**–**iii**)). Emission spectra (**i**), intensity (**ii**), and lifetime (**iii**) were measured for 12 cycles, lasting about 3 min each.

The reversibility of the ion response was tested by immersion of each lens in a 0.0 mM or high concentration (130 mM Na^+^ or 110 mM K^+^). Both the intensities and lifetimes were found to be completely reversible over multiple cycles (Figure 8). Both probes were tested for washout of the contact lenses. These tests were performed on both the parent probes without PL and with the PL-bound probes. These results showed that washout did not occur for SG-PL or PG-PL, but some washout occurred for SP-PL for the probes SG and PG without PL (Figures S5 and S6). The lack of probe washout and the low amounts of probe bound to the lenses suggest that there will be no toxic effects on the cornea if worn by patients.

**Figure 9:**
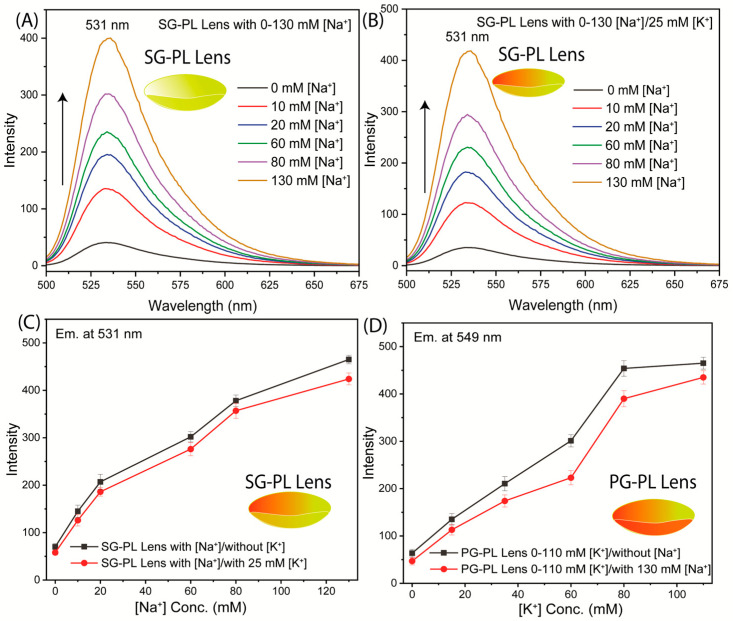
SG-PL Lens with different concentrations of Na^+^ ions with or without 25 mM K^+^ ions (**A**,**B**), and corresponding emission intensity differences (**C**). PG-PL lens with different concentrations of K^+^ ions with or without 130 mM Na^+^ ions, and corresponding emission intensity difference (**D**).

Another important consideration is the interference of K^+^ measurements with the Na^+^ measurement. Interference by sodium is expected to be the dominant effect because the smaller Na^+^ ion can fit into the larger azacrown for K^+^ (Figure 2). There was no interference effect on the Na^+^ measurement due to high K^+^ concentration (Figure 9). A minor interference effect was observed for measurement of K^+^ in the presence of high Na^+^ concentration (Figure 9D). This minor effect is not likely to affect the K^+^ measurements because the high Na^+^ concentration varies over a narrow range compared to its total concentration in tears.

**Figure 10:**
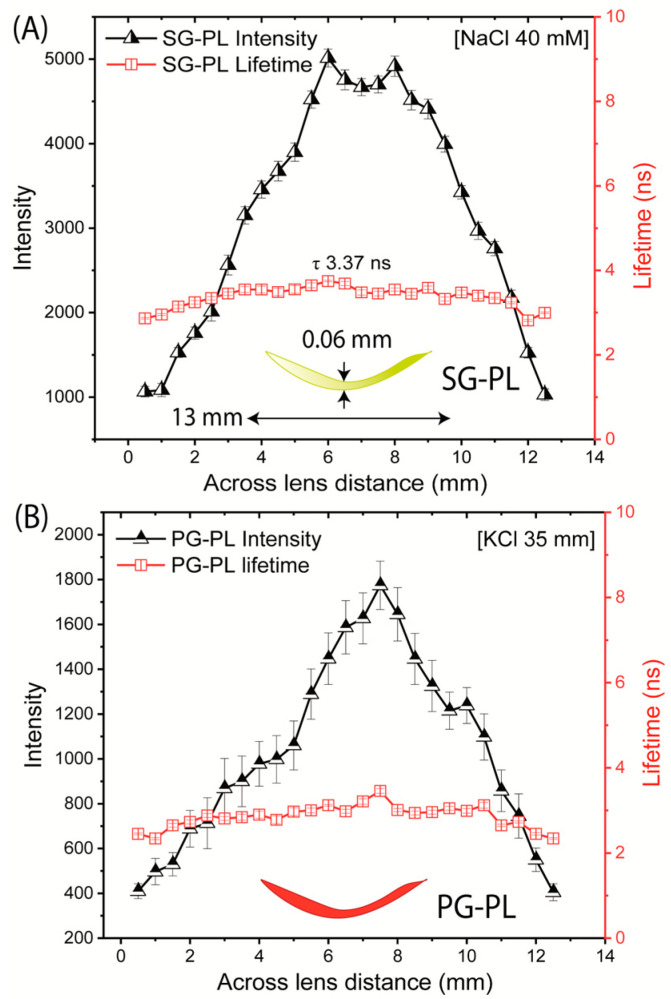
Fluorescence intensities and lifetimes measured across the SG-PL (**A**) and PG-PL (**B**) Lot A lenses (13 mm length).

We evaluated the probe distribution in the lenses and the lifetimes across the lenses. As expected, the intensity was highly variable depending on location (Figure 10). The intensity of each probe in the lens decreased to zero when the focal spot was off the edge of the lens. The intensity changes because the confocal spot of this was not adjusted to remain directly on the curved surface of the lens. In contrast to the intensities, the lifetimes of SG-PL via PG-PL were constant across the entire lens (Figure 10). The result suggests that lifetime-based lensing is possible with a labeled contact lens.

**Figure 11:**
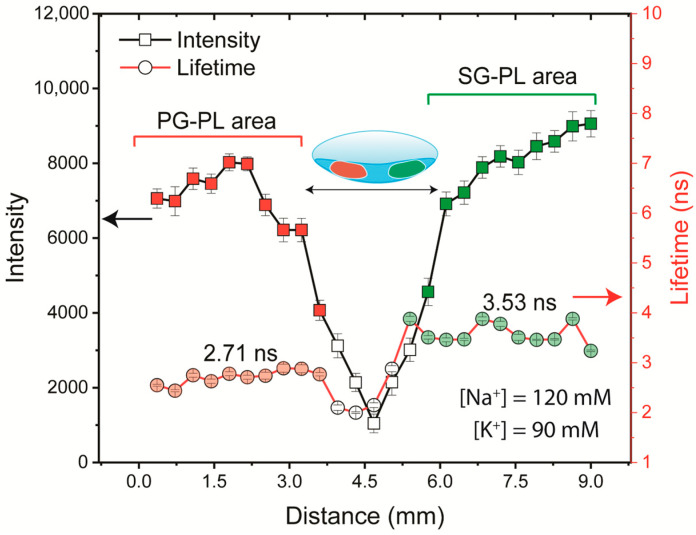
Detection of Na^+^ and K^+^ using a single Lot A lens using intensity or FLIM measurements. The lens was scanned across a 10 mm length (Step size 0.36 mm), measuring the intensity and lifetime of probes. The lens was labeled on the left side with PG-PL and on the right side with SG-PL.

As a final test we placed all three probes in a single lens and recorded the emission spectra and wavelength-dependent lifetimes at various concentrations of Na^+^ and K^+^. However, the spectral overlap of the probes prevented the separate detection of the Na^+^ and K^+^ concentrations. We then proceeded with the option of separate locations of the probes on the lens. Because both the lens and probe solution were hydrophilic, the spots from a micropipette spread rapidly. Hence, we then carefully dipped the edge of the lens into either a solution of SG-PL or PG-PL. The procedure provided a lens with one-half containing each probe and a blank region in the center (Figure 11). The intensity and lifetime measurements clearly showed the different spectral properties on each half of the lens. This result demonstrated that a successful NaK-CL contact lens can be made with the three probes used in this report.

## 4. Discussion and Conclusions

There are many advantages to the use of contact lenses for use in medical diagnostics and research. The present report on the NaK-CL lens represents only the first step in a longer-term development of NaK-CL lenses and fluorescent contact lenses for other sensing applications. We acknowledge that its design is not ideal. The NaK-CL can be improved in several ways. For example, there is excessive overlap of the probes' emission spectra, which partially diminishes the value of the RhB reference fluorophore. The difficulty can be easily overcome by a longer wavelength rhodamine derivative [48,49,50]. The emission spectra of SG-PL and PG-PL display too much overlap for simple independent measurements. It is highly likely that similar Na^+^ and K^+^ dependent probes can be developed, and useful structures are already known [51,52,53,54]. Additionally, the lifetimes of the three probes used in this report are too similar, and a larger difference between the values would more easily allow lifetime-based sensing to minimize the effects of patient motion.

There is an alternative approach to avoid the problems described above, which is to have the probes spatially separated in the lens. We have already demonstrated that the PL-linked probes do not migrate across the lens and that the PL-linked probes do not diffuse through the lens from the front to back surface [55]. The lenses, by design, are wet and have a hydrophobic surface. As a result, our early attempts to label spots were not successful and resulted in poorly localized probes. A large amount of research has been devoted to localizing spotting of similar solutions on hydrophilic surfaces [56,57,58], and we are presently working on this topic.

There are other considerations for the clinical use of labeled contact lenses. Of course, the lenses must be tested for toxicity, in particular effects on the sensitive corneal layer. The probes used in the presence of NaK-CL are similar to those already in use in ophthalmology, such as fluorescein and Indocyanine green, and the amount of probes in the lenses is much less than that used for corneal staining. Additionally, we found that the PL-linked probes do not leach out of the lenses.

The NaK-CL will be simple to use in clinical practice. We have spoken with many individuals about their first use of contact lenses, some with HG lenses and the majority with SiHG lenses. For a large majority of the individuals, the initial insertion of a lens was rapid and painless, and none were reported to use an ocular anesthetic. The same ease-of-use results were found for most new CL users. We questioned the former chairman of Ophthalmology at the University of Maryland, Baltimore, USA. He stated that he has never encountered a patient who did not readily adapt to the insertion of a SiHG lens, even patients who have never worn CLs.

New CL users adapted very quickly to their new lenses. The patients expressed a few minutes of blurred vision until the lens became centrally located in the eye. A small number of patients had some eye irritation for the first several days, which quickly resolved, sometimes with eye drops. The conclusions from this report are that patients above 4 years of age can wear sensing contact lenses in a hospital setting. Many of the elderly patients have previously worn contact lenses but later found regular glasses more convenient. The NaK-CL lenses will be fabricated without optical correction so the patient can continue with their present glasses for distance, reading, or bifocals. Patients who require frequent blood draws will be happy to have a reduced number by using the NaCl lens.

Continuous vs. on-demand ion concentration measurements: The type of measurements will depend on the design of the optical reader and the clinical requirements, and different devices can be designed for each type of measurement. The incident light does not need to be directed into the eye but can pass across the lens like the currently used slit lamps. For a NaK-CL the incident interaction will be much lower than a slit lamp. Given the current advanced state of electronics technology and the variety of optical detectors reported for vision, there is no doubt that suitable devices could be developed. The CL readers could be a hand-held device, such as a cell phone, or a pair of glasses with built-in light sources and detectors, as shown in Figure 1. The vast knowledge from medical and defense applications (such as LIDAR) for remote sensing ensures that the needed devices can be designed and constructed. At present, due to the limited resources for research funding and absence of the infrastructure needed for electronic design, we focused on the ion-sensing probes and measurement methods.

Response time of measurements: There are several different factors to consider in the response time of the NaK-CL lens. The time response reported by the NaK-CL lens is likely to be closely related to the turnover time from the lachrymal duct secretions. The rate of tear turnover rate is near 17% per minute [59], so the time to replace the tear volume is near 5 min. The tear flow rate will typically increase when a lens is placed on the eye, which changes the tear composition. These effects typically return to the basal rate in 10 min or less. The NaK-CL can be worn as long as needed for the tears to return to the hard level composition.

The response time of the NaK-CL can also be affected by the pathway of tear flow. Tears can flow around, over, and under the CL. Flow around the lens may not affect the optical reading. Tear flow over the CL occurs with the pre-lens tear film (PLTF), and tear flow under the lens and between the lens and cornea occurs in the post-lens tear film (PoLTF). The PoLTF is about 10-fold thinner than the PLTF and is expected to occur more slowly. Washout measurements of fluorescence dyes do not distinguish between these two film layers, but the multiple time constants indicate a slower component likely to be due to the PoLTF. These different flow paths and time constants could affect the time response of the NaK-CL. If diffusion of ions through the lens is rapid, then both values will remain the same. Presently there is little information on ion transport rates through the pores of the lens. It is not practical to calculate time constants for these complex channels. Fortunately, there is a way to eliminate this transport effect, or if needed, to measure exchange rates in the PLTF and PoLTF. We recently reported that Na^+^ and K^+^ dyes could be bound in the inner or outer layer of a CL, and the probes do not diffuse across the lenses for a period of days to weeks. If necessary, a NaK-CL can be placed on just the outer surface of the lens, in the PLTF, for detection in the faster flow region.

Errors in [K^+^] measurements: It is known from panel testing that the [K^+^] measurements often have greater uncertainty than the [Na^+^] measurements. This is thought to result from the higher intracellular [K^+^] in cells near 150 mM as compared to the [K^+^] in blood near 4 mM and in tears near 24 mM (Table S1). Therefore, any cell damage during a blood draw will cause the K^+^ measurements to be elevated in the blood panel. This effect is not expected to be a problem with the NaK-CL because there are few cells in tears, and the measurements can always be delayed till tear exchange is complete.

Cost of the NaK-CL lenses: At present there are many research programs to develop sensing contact lenses [59,60,61,62]. Almost all these efforts are focused on known electrical and sensing measurements, such as ion-selected electrodes with the size reduced to fit with the contact lenses. A sample of these publications reveals a dependence on known sensing chemistry with reduced size to fit with a CL. Such opto-electronics contact lenses (OE-CLs) will almost certainly be more expensive than our proposed lenses. Additionally, the surface chemistry of SiHG lenses has been evolving rapidly, and this could increase the costs of new OE-CL. The emphasis of OE-CL is likely the result of research projects located in engineering departments rather than biophysical chemistry and/or fluorescence laboratories. The research costs for fluorescent Na and K sensors for use in lenses are likely to be much less than the cost to develop a new OE-Cl.

Ethical considerations: The clinical testing of the NaK-CL lenses appears to be more straightforward than a new drug. The NaK-CL lenses can be tested for accuracy and safety without any significant risk to the patients. Almost all elderly patients admitted to the hospital will be subjected to an EB or BMP test, which is the current standard of medical care. The additional use of a NaK-CL will provide additional information about the patient's Na^+^ and K^+^ concentration.

The measurements for the NaK-CL results can be used or not used to decide on further treatment, or can be used to follow ion concentration levels during rehydration treatment. Neurological damage can occur if rehydration is too rapid. Many of the SiHG lenses are FDA-approved for 30 days of continuous wear, even while sleeping, because of high oxygen transport. The longer-term wear of the SiHG lens reduces the possibility of contaminants during insertion or deletion of the lenses. The present results on the possibility of a NaK-CL are likely to stimulate further development of this opportunity.

## Data Availability

Data supporting the findings of this study are available from the corresponding author Joseph Lakowicz upon request.

## Supplementary Materials

The following supporting information can be downloaded at https://www.mdpi.com/article/10.3390/bios15080532/s1, Figure S1: Schematic of the nanoporous contact lenses, Table S1: Electrolyte concentrations in serum, tears, and cells, Figure S2: Lens’s uptake of SG-PL and PG-PL probe, Figure S3: The absorbance spectra, Figure S4 and S5: Measurement of probe washout of SG and PG, Figure S6: Measurement of probe washout of PG (i) and PG-PL (ii) from a Com A lens. Summary values are in (iii), and Table S2: Summary of the Present Work.
