# Supplementary material for: First Design of a Contact Lens for Diagnosis of Dehydration
Source: Biosensors (Basel). 2025 Aug 14;15(8):532. doi: 10.3390/bios15080532 (PMC12384969; doi:10.3390/bios15080532)
Supplement: Supplementary file 1 [file biosensors-15-00532-s001.zip › biosensors-3720911-supplementary.pdf]

Supplementary Materials

First Design of a Contact Lens for Diagnosis of Dehydration

Kundan Sivashanmugan [1], Reece E. Albert [2] and Joseph R. Lakowicz [1,*]

[1] Center for Fluorescence Spectroscopy, Department of Biochemistry and Molecular Biology, University of Maryland School of Medicine, 655 W. Baltimore St, Baltimore MD 21201, USA

[2] Department of Obstetrics, Gynecology and Reproductive Sciences, University of Maryland School of Medicine, 655 W. Baltimore St, Baltimore, MD 21201, USA

* Correspondence: jlakowicz@som.umaryland.edu

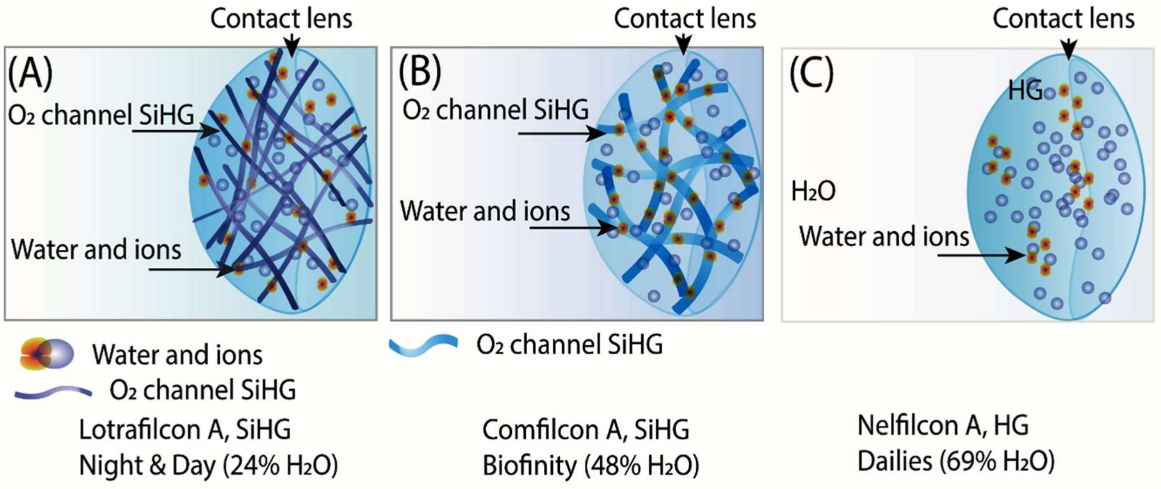

Polymer	Trade Name	Manufacture	Wear Days	Water %	Dk (at -3.00D)
Lotrafilcon A (SiHG)	Air Optix	Alcon	30	24	175
Comfilcon A (SiHG)	Biofinity	Cooper Vision	30	48	160
Nelfilcon (HG)	Dailies	Ciba Vision	1	69	26

Figure S1. Schematic illustration of contact lenses with a nanoporous network: (A) Lotrafilcon A silicone hydrogel (SiHG), (B) Comfilcon A SiHG, and (C) Nelfilcon hydrogel (HG). Bottom table shows contact lens details. Dk represents the diffusion coefficient (D) and the solubility constant (k). The lenses are drawn with silicone or water channels based on their fractional contents but we do not know the actual topology of the lenses.

Table S1. Electrolyte Concentrations in Serum, Tears, and Cells.

Ions	Serum	Tears	Cells

**pH**	7.35 – 7.46	6.5 – 7.6	7.4
**H^+**	35 – 45 nM	25 – 52 nM	0-40 nM
**Na^+**	135 – 145 mM	132 mM	10 mM
**K^+**	3.5 – 5.3 mM	24 mM	150 mM
**Ca^{2+}**	4.5 – 5.5 mM	0.8 mM	0.8 mM
**Mg^{2+}**	0.7 – 1.0 mM	0.6 mM	15 mM
**Cl^-**	96 – 110 mM	118 – 138 mM	

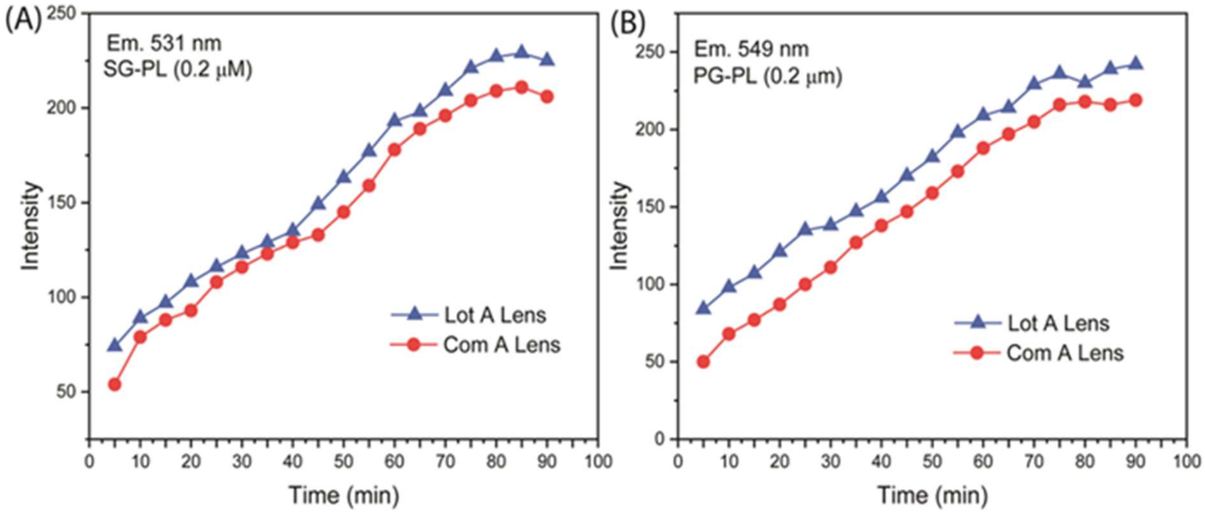

Figure S2. Lens uptake SG-PL (A) and PG-PL (B) probe with 5-minutes in the probe solutions for Com A and Lot A lenses.

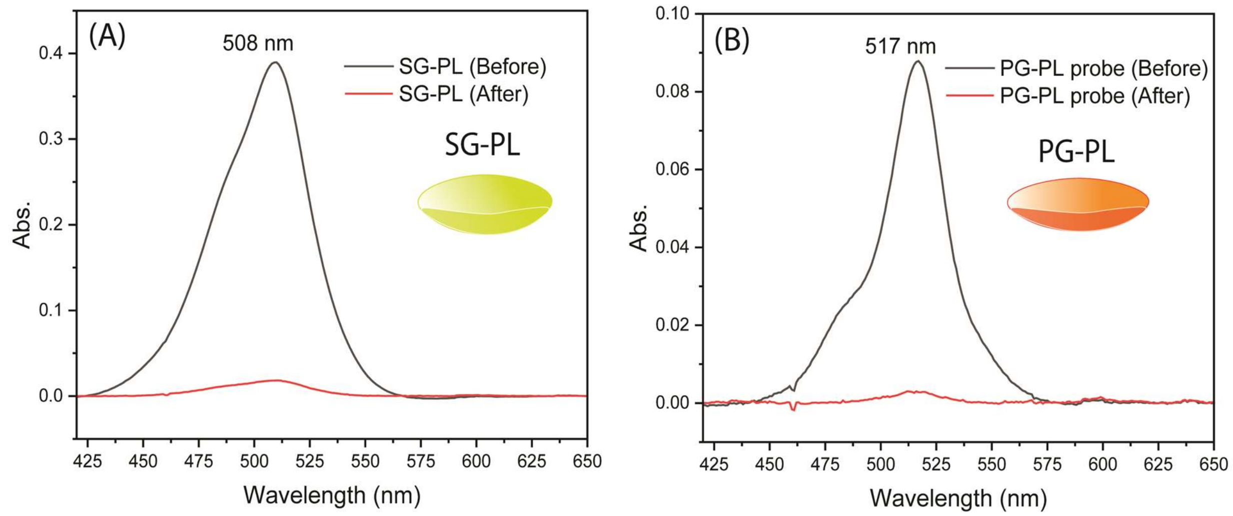

Figure S3. The absorbance spectra were obtained before and after lens uptake solutions (A) SG-PL and (B) PG-PL probes before and after 12 dip and wash cycles. The ion concentrations 120 mM and 46 mM, for Na and K, respectively.

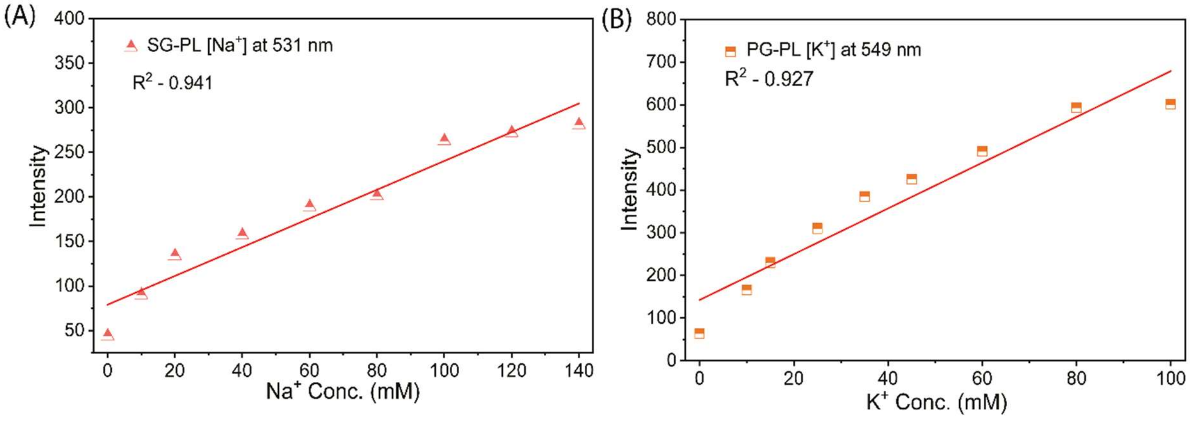

Figure S4. Linear relationship between fluorescence intensity and Na^+ (0-140 mM) (A) and K^+ (0-100 mM) (B) concentrations.

The sodium-sensitive green contact lens detected Na^+ ions at concentrations ranging from 10 mM to 140 mM. Notably, the lens demonstrated potential sensitivity below the millimolar level. Figure S4 shows that fluorescence intensity increased linearly with Na^+ concentration. The

response was stronger in the low concentration range (0-40 mM), with a continuous rise at higher values (>100 mM). The current experimental setup exhibited a minimal detection limit of 10 mM, which is within the physiological sodium range. The potassium-sensitive green contact lens can detect K^+ concentrations within the physiological range. The response was stronger in the low concentration range (0-35 mM), with a continuous rise at higher values (>100 mM). The approach was primarily qualitative rather than quantitative in this work. Although a qualitative increase in fluorescence intensity was observed with increasing Na^+ or K^+ concentration, saturation of the contact lens surface was not achieved. This is likely due to the continuous uptake of Na^+ or K^+-sensitive probes by the lens material, making it challenging to reach a saturation point after probe incubation. Both probes demonstrated sensitivity within the physiological concentration ranges of these ions.

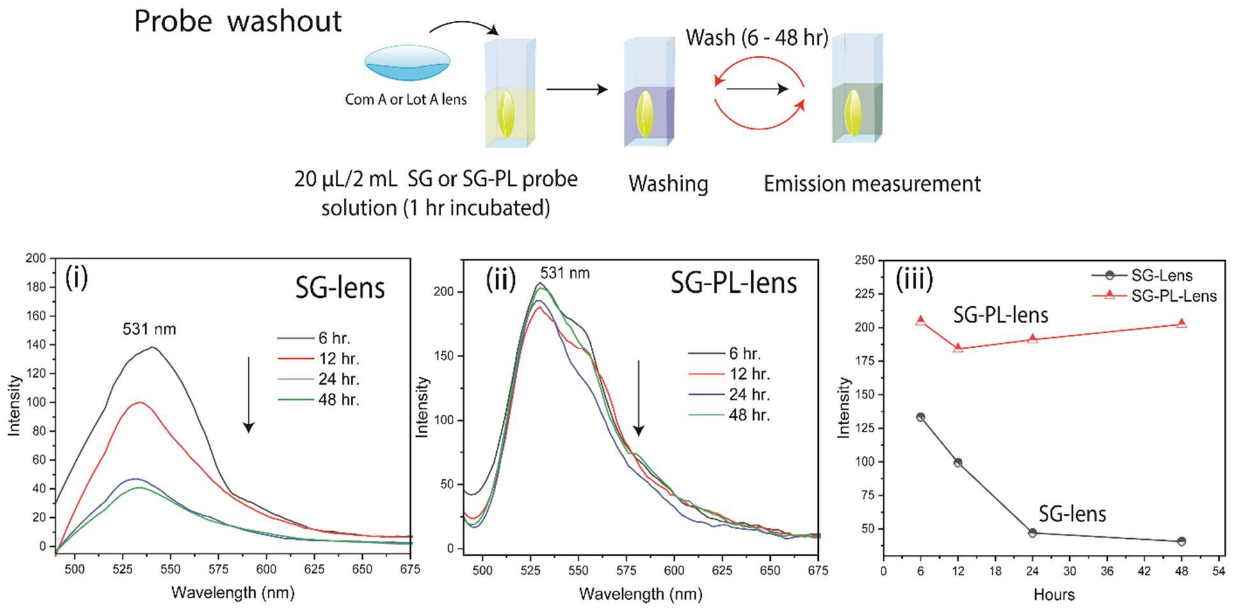

Figure S5. Measurement of probe washout of SG(i) and SG-PL(ii) from a Com A lens. Summary values in (iii).

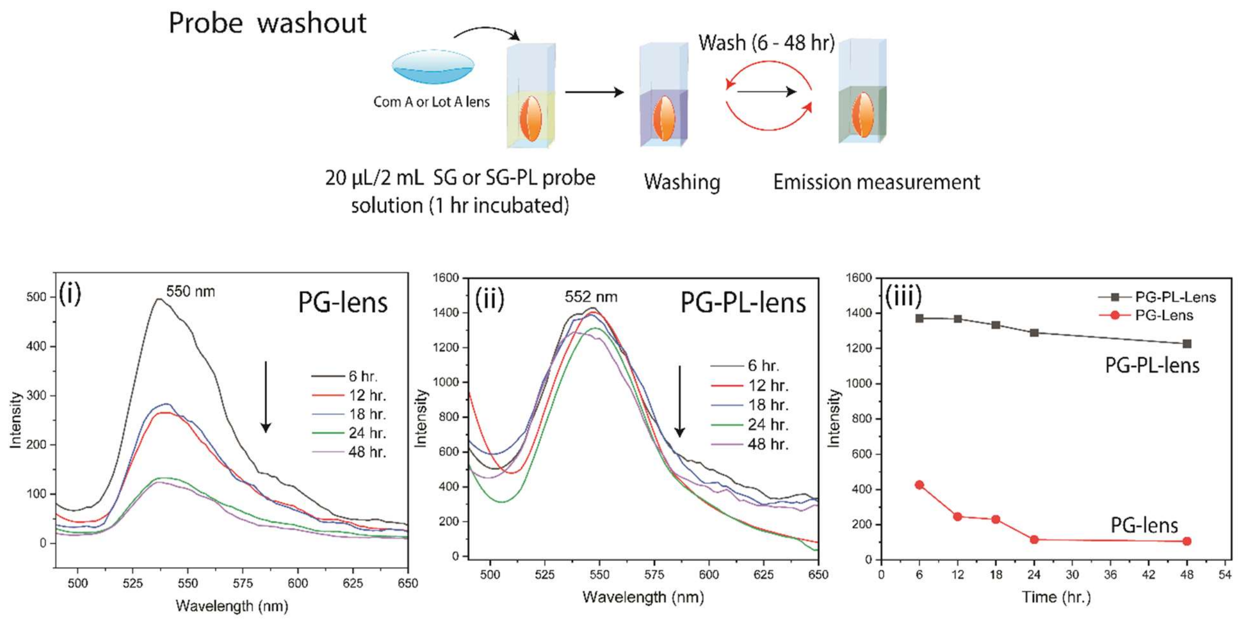

Figure S6. Measurement of probe washout of PG (i) and PG-PL (ii) from a Com A lens. Summary values are in (iii).

Table S2. Summary of the Present Work.

**Materials**	**Technology Used for Measurement**	**Possible Detection Sample**	**Advantages of Present Work**	**Disadvantages**
-Commercial SiHG contact lenses<br><br>- Other SiHG	- Fluorescence emission<br><br>- Wavelength ratio<br><br>- Probe lifetime<br><br>- Emission imaging	- Tear samples<br><br>- Purified<br><br>biological fluids	- Rapid uptake of tear samples<br><br>- Requires low tear volume<br><br>- Multiple ion detection<br><br>- No electrical components needed<br><br>- Non-toxic probes	- Tear collection is difficult in patients with dry eye disease
